# Cancer registry study of malignant hepatic vascular tumors: hepatic angiosarcomas and hepatic epithelioid hemangioendotheliomas

**DOI:** 10.1002/cam4.4403

**Published:** 2021-12-01

**Authors:** Constanza Martínez, Jonathan K. Lai, Daryl Ramai, Antonio Facciorusso, Zu‐Hua Gao

**Affiliations:** ^1^ Department of Radiation Oncology McGill University Montreal Quebec Canada; ^2^ Department of Pathology McGill University Montreal Quebec Canada; ^3^ Department of Medicine The Brooklyn Hospital Center Brooklyn New York USA; ^4^ Division of Gastroenterology & Hepatology CHI Health Creighton University Medical Center Omaha Nebraska USA; ^5^ Division of Gastroenterology University of Nebraska Medical Center Omaha Nebraska USA; ^6^ Division of Gastroenterology Moffitt Cancer Center University of South Florida Tampa Florida USA; ^7^ Section of Gastroenterology Department of Medical Sciences University of Foggia Foggia Italy

**Keywords:** hepatic angiosarcomas, hepatic epithelioid hemangioendotheliomas, hepatic malignant vascular tumors, prognosis, SEER, surgery

## Abstract

**Background:**

Malignant vascular tumors (MVTs) are rare and often misdiagnosed due to wide range of clinical presentations, varied histology, and exquisite imagining features. We aim to characterize two different types of MVTs of the liver: hepatic angiosarcomas (HA) and hepatic epithelioid hemangioendotheliomas (HEHE).

**Methods:**

Data on HA and HEHE between 1975 and 2016 were extracted from the SEER database and analyzed.

**Results:**

A total of 366 patients with HA were identified where 64.2% were male and 79% of White race. The median age at diagnosis was 64 ± 16.2 years. Distant metastasis was found in 24% of patients, regional disease in 22.1%, and localized disease in 21.3%. The median overall survival for HA was 2 months. For HEHE, 120 cases were identified, 32.5% were male and 80% of White race. The median age of diagnosis was 51 ± 16.8 years. Distant metastasis was found in 37.5% of patients, regional disease in 27.5%, and localized disease in 20%. The median overall survival was 182 months.

**Conclusion:**

Patients’ demographics such as race, age, and gender may assist in elucidating distinct subtypes of MVTs. HA is an aggressive tumor despite intervention. Patients with HEHE tumors have significantly better survival compared to patients with HA. Further studies are needed to deepen our knowledge about the histopathology of these tumors, the outcomes of liver transplantation as a therapeutic alternative, and available molecular targets for MVTs.

## Graphical abstract

In the current manuscript, we performed a retrospective analysis using the Surveillance, Epidemiology, and End Results Registry (SEER) database to have the most updated characterization of the two most prevalent histological malignant hepatic vascular tumors; angiosarcomas and epithelioid hemangioendotheliomas. Our data includes a review of the literature, histopathological characterization of the malignancies with high‐quality images, current therapeutic alternatives for these types of cancer, and highlights of the missing and unmet clinical needs when conducting studies about hepatic malignancies.

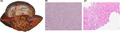

## 1 INTRODUCTION

Mesenchymal tumors of the liver encompass a wide variety of common and uncommon hepatic neoplasms arising from the non‐epithelial hepatic compartment, which includes soft tissue tumors, gastrointestinal stromal tumors, hemangiomas, granular cell tumors, schwannomas, and perineuromas. The most common subset of mesenchymal tumors is benign hemangiomas. Malignant vascular tumors (MVTs) are a less common group of tumors that are derived from endothelial cells of the liver.^1^ The most prevalent MVTs are hepatic angiosarcomas (HA) and hepatic epithelioid hemangioendotheliomas (HEHE).^2^ MVTs are often misdiagnosed entities due to their wide clinical presentation, varied histology, and imagining features. These tumors are mostly identified incidentally on radiological studies and ultimately confirmed by histology and immunohistochemistry.^3^, ^4^

**FIGURE 1:**
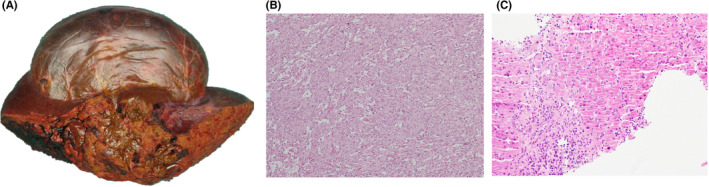
Hepatic angiosarcoma. A. Gross image showing the tumor bulges out from the capsule and extends deep into the parenchyma, with extensive hemorrhage and necrosis. B. Solid area shows atypical tumor cells and anastomosing vascular channels (H&E 100x). C. Tumor cells line the sinusoids and invade into portal tracts and portal vein (Left H&E 100x)

Primary HA represents 2% of all liver neoplasms and accounts for the most common type (9.4%) of MVT.^2^, ^5^, ^6^ HA is more prevalent in men in their 70s and its pathogenesis has been associated with exposure to environmental chemicals such as thoroplast, arsenic, and vinyl chloride.^7^ On imaging, these vascular lesions often demonstrate large, solid, or cystic lesions. Histologically, the tumor is composed of atypical, mitotically active spindled, or epithelioid endothelial cells with a sinusoidal, scaffold‐like, trabecular, pseudo papillary, or cavernous growth pattern (Figure 1A–C **)**.^8^ Genomic studies have revealed common molecular alterations of HA tumors. Whole genome sequencing has characterized genomic alteration in HSPG2 (heparan sulfate proteoglycan 2), COL1A1 (Collagen type 1 alpha 1 chain), BCRA1 and BRCA2 frameshift deletions, and a recurrent PAGE1 mutation.^9^ The standard of care for HA is still surgery and adjuvant therapy.^10^ This has not been modified or tailored in a personalized manner. Therefore, the overall survival (OS) for treated patients is approximately 6 months and as low as 1 month. This poor OS is mostly explained due to complications such as intraperitoneal hemorrhage that rapidly contributes to clinical deterioration.^5^, ^11^, ^12^

**FIGURE 2:**
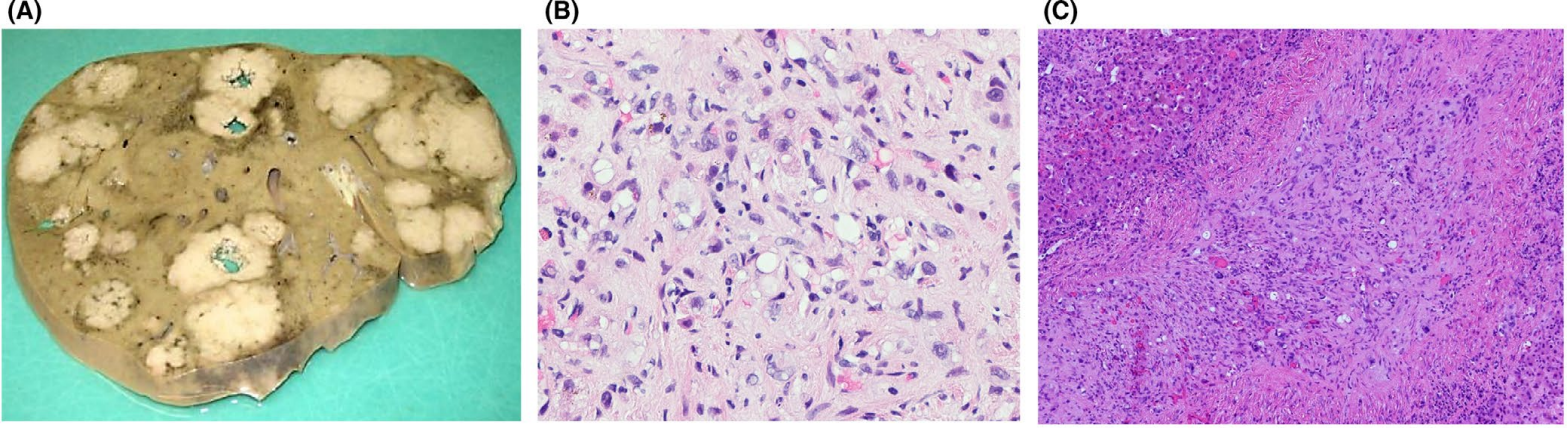
Hepatic epithelioid hemangioendothelioma. A. Gross image showing multifocal large, well circumscribed, tan‐white masses. B. Histological image showing atypical epithelioid cells with intracytoplasmic vacuoles and spindle cells in myxoid stroma (H&E 200x). C. Atypical tumor cells grow within the lumen of a large hepatic vessel (H&E 100x)

HEHE are very rare (1 in a million) MVTs with a predominance in females in their fourth decade of life.^13^, ^14^ It can present either indolently or with multiple metastases at the time of diagnosis. It often appears as multifocal solid lesions and involves various organs such as liver, lung, and spleen, hence, it is also often misdiagnosed as benign pathologies or cofounded with liver metastasis (Figure 2A–C **)**.^15^, ^16^ On imaging studies, HEHE shows characteristic “target sign” and “lollipop sign.” The “target sign” is composed of an inner ring as defined by a fibrotic center, a middle ring as defined by a zone of epithelial proliferation, and an outer ring as defined by a narrow peripheral avascular zone between the nodules and liver parenchyma. The “lollipop sign” is due to the tumor spread via the portal and hepatic vein branches, which consists of a well‐defined tumor mass and the adjacent occluded vein simulates the lollipop stick on enhanced images.^17^ Moreover, tumor cells can grow along the lumen of existing large vessels and later cause vascular obstruction leading to the clinical presentation mimicking Budd–Chiari syndrome.^18^ Some studies suggested an association between Bartonella henselae infection and HEHE.^19^ Molecular genomic studies in these tumors have identified characteristic t(1;3)(p36;q25) translocations resulting in WWTR1/CAMTA1 fusion protein^20^, ^21^ and t(11;X)(q13;p11) translocations resulting in YAP‐TFE3 fusion proteins.^20^, ^21^, ^22^ HEHE does not have standardized therapeutic options. Liver transplantation (LT) is the most common treatment available. However, surgery, adjuvant treatments, and radiation therapy are also offered to these patients.^16^, ^23^ The 1‐ and 5‐year overall survival are 54% and 24%, respectively.^2^ Multicentric studies and molecular characterization of pathogenic genomic alterations are in need to identify optimal therapeutic strategies for HEHE.

Advancement in imaging technologies has increased the diagnostic sensitivity and specificity of MVTs. Targeted therapies as the result of molecular genetics studies have offered new hope of cure for these devastating vascular malignancies of the liver. In this context, it is important to update our knowledge on the demographics and clinical behaviors of MVTs. This article provides the most updated analysis to date using the SEER database––a national cancer registry. In this study, we examined the prevalence, demographics, tumor features, treatments, and outcomes of patients with HA and HEHE. We also created survival prediction models to determine prognosis that may aid clinical decisions.

## 2 PATIENTS AND METHODS

Data regarding patients diagnosed with HA and HEHE from 1975 to 2016 were extracted from the Surveillance, Epidemiology, and End Results Registry ^(SEER).^ ^24^ As defined by the International Classification of Diseases for Oncology, 3rd edition (ICD‐O‐3), the morphological codes used for HA and HEHE were 9120 and 9130, respectively.^25^ Data were collected from 18 registries [San Francisco–Oakland, Connecticut, metropolitan Detroit, Hawaii, Iowa, New Mexico, Utah (since 1973), Seattle–Puget Sound (since 1974), metropolitan Atlanta (since 1975), Alaska, San Jose–Monterey, Los Angeles, rural Georgia (since 1992), greater California (excluding San Francisco, Los Angeles, and San Jose), Kentucky, Louisiana, New Jersey, and greater Georgia (excluding Atlanta and rural Georgia, since 2000)], which, in aggregate, represents nearly 28% of the US population (1,2).

The demographic variables of interest were patient sex, age at diagnosis, race, and year of diagnosis. Race was recorded as White, Black, American Indian/Alaska Native, and Asian or Pacific Islander. The year of diagnosis was treated as a continuous variable from 1975 to 2016. Clinical variables included disease‐specific survival (DSS) and treatments. Therapeutic interventions were divided into surgery, radiation, and chemotherapy. Pathological information included the tumor size, tumor stage, and tumor grade. Since the AJCC data is missing in a significant proportion of patients, a SEER summary staging was used. Tumor size was treated as a continuous variable.

A complete‐case method was used for missing data. Revision of the data showed no evidence of systematic association between the missing data and any of the independent and dependent variables in the study. Thus, the assumption that missing data was completely at random was reasonable and the possible effects of selection bias was minimal.

### 2.1 Statistics

Survival was calculated for primary HA and HEHE. SEER*Stat statistical software was used to estimate cause‐specific survival. Cause‐specific survival was defined as net survival measure representing survival of patients with HA and HEHE in the absence of other causes of death. Individuals who die of causes other than those specified are censored. Cox proportion hazard (PH) regression modeling was used to determine predictors of survival. Cox PH assumptions were evaluated by examining Schoenfeld residuals. Cox PH models were true if the hazard was reasonably constant over time.

SEER*Stat statistical software was also used to perform adjusted frequencies and rate statistics (1,2). IBM SPSS Statistics was used for Cox proportional hazard regression modeling to determine predictors of survival. Cases in which survival duration was unknown were excluded and statistical significance was set at *p* < 0.05. SEER data are publicly available, and all patient information is de‐identified; therefore, the current study was deemed to be exempt from institutional review board approval.

## 3 RESULTS

### 3.1 Demographics

We identified 366 patients with HA (64.2% males and 35.8% females) and 120 patients with HEHE (32.5% male and 67.5% females). The median age at diagnosis was 64 ± 16.2 years for HA and 51 ± 16.8 years for HEHE. MVTs showed a predilection toward White race (79% HA and 80% HEHE), followed by Asian or Pacific Islander (15.8% in HA and 1.7% in HEHE) (Table 1).

**TABLE 1:** Study characteristics

Variables	HA (*n* = 366)	HEHE (*n* = 120)
Sex		
Male	235 (64.2%)	39 (32.5%)
Female	131 (35.8%)	81 (67.5%)
Race		
White	289 (79.0%)	96 (80.0%)
Black	17 (4.6%)	13 (10.8%)
American Indian/Alaska Native	2 (0.5%)	9 (7.5%)
Asian or Pacific Islander	58 (15.8%)	2 (1.7%)
Unknown		
Tumor grade		
Well differentiated; Grade I	8 (2.2%)	4 (3.3%)
Moderately differentiated; Grade II	12 (3.3%)	11 (9.2%)
Poorly differentiated; Grade III	37 (10.1%)	5 (4.2%)
Undifferentiated; anaplastic; Grade IV	37 (10.1%)	1 (0.8%)
Unknown	272 (74.3%)	99 (82.5%)
SEER stage		
Localized	78 (21.3%)	24 (20.0%)
Regional	81 (22.1%)	33 (27.5%)
Distant	88 (24.0%)	45 (37.5%)
Unknown	119 (32.5%)	18 (15.0%)
Surgery		
Surgery performed	52 (14.2%)	33 (27.5%)
Surgery not performed	314 (85.8%)	87 (72.5%)
Radiation		
Received radiation	18 (4.9%)	10 (8.3%)
Did not receive radiation	348 (95.1%)	110 (91.7%)
Chemotherapy		
Received chemotherapy	112 (30.6%)	41 (34.2%)
Did not receive chemotherapy	254 (69.4%)	79 (65.8%)

Abbreviations: HA, hemangiosarcoma; HEHE, hepatic epithelioid hemangioendothelioma.

### 3.2 Characteristics

For patients diagnosed with HA, most tumors were characterized as undifferentiated (10.1%), followed by poorly differentiated (10.1%), moderately differentiated (3.3%), and well differentiated (2.2%). For patients diagnosed with HEHE, most tumors were characterized as moderately differentiated (9.2%), followed by poorly differentiated (4.2%), well differentiated (3.3%), and undifferentiated (0.8%). Unknown tumor grade accounted for 74.3% and 82.5% of HA and HEHE cases, respectively.

Using SEER staging criteria, most HA tumors were characterized as distant (24%), followed by regional (22.1%) and localized (21.3%). For HEHE, most tumors were also characterized as distant (37.5%), followed by regional (27.5%) and localized (20%). HA tumors had a median size of 163 ± 488.8 mm compared to HEHE which had median sizes of 90 ± 468 mm.

### 3.3 Treatment

Most tumors were not surgically resected. About 85.5% of HA tumors were not resected. Similarly, 72.5% of HEHE did not undergo surgical resection. Only 4.9% of HA patients received radiation therapy compared to 8.3% of HEHE who received radiation therapy. About 30.6% of HA patients received chemotherapy. Similarly, 34.2% of HEHE patients received chemotherapy.

### 3.4 Survival and clinical predictors

**FIGURE 3:**
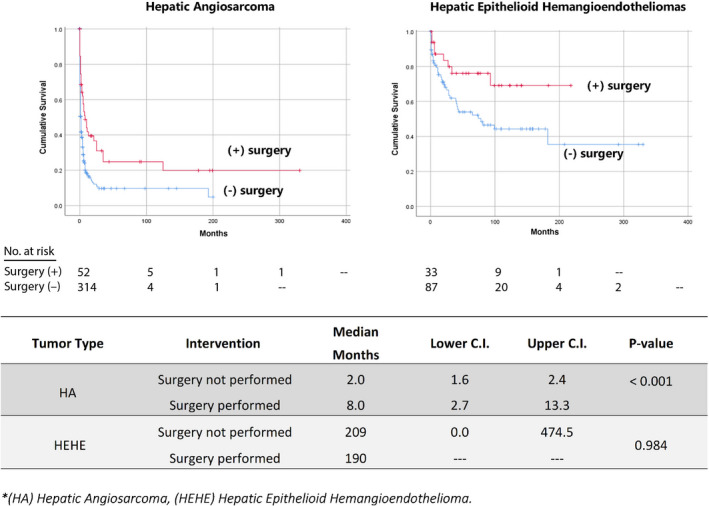
The Kaplan–Meier estimations of hepatic angiosarcomas and hepatic epithelioid hemangioendothelioma

Overall, 1‐ and 5‐year cause‐specific survival for HA were 19% and 14%, respectively, with a median survival of 2 months. The 1‐ and 5‐year cause‐specific survival for HEHE were 72% and 59%, respectively, with a median survival of 182 months (Table 2). We performed the Kaplan–Meier estimations of patients who had surgical resection. For patients with HA, the median survival of patients who had surgical resection was 8 months compared to 2 months for patients who did not have surgery (*p* < 0.001). For patients with HEHE, median survival of patients who had surgical resection was 190 months compared to 209 months for patients who did not have surgery (*p* < 0.984) (Figure 3).

**TABLE 2:** Survival characteristics

	HA	HEHE
Cases	366	120
Age (Median, SD)	64 ± 16.2	51 ± 16.8
Tumor size in mm (Median)	163 ± 488.8	90 ± 468.0
Cause‐specific		
1 year	19%	72%
5 year	14%	59%
Median	2 months	182 months

Abbreviations: HA, hemangiosarcoma; HEHE, hepatic epithelioid hemangioendothelioma.

On Cox proportional hazard regression analysis, for patients diagnosed with HA, significant outcomes were noted among patients with distant tumors (hazard ratio [HR] 1.92, 95% confidence interval [CI] 1.19–3.10, *p* = 0.008), treated with surgery (HR 0.50, 95% CI 0.26–0.96, *p* = 0.036), treated with radiation (HR 0.394, 95% CI 0.16–1.00, *p* = 0.049), and treated with chemotherapy (HR 0.33, 95% CI 0.21–0.51, *p* < 0.001). No significant predictors of mortality were noted on Cox proportional hazard regression analysis for HEHE regarding their stage and treatment (Table 3).

**TABLE 3:** Cox regression analysis

	HA			HEHE		
Characteristics	Hazard ratio	95% CI	*p* value	Hazard ratio	95% CI	*p* value
Sex						
Male	1.00			1.00		
Female	0.75	0.51–1.11	0.153	0.73	0.33–1.62	0.437
Age	1.02	1.00–1.03	0.010	1.01	0.98–1.04	0.458
Tumor size	1.00	1.00–1.00	0.018	1.00	1.00–1.00	0.397
Race						
White	1.00			1.00		
Black	1.30	0.63–2.69	0.476	2.93	0.66–13.08	0.159
Asian or Pacific Islander	1.12	0.72–1.75	0.610	1.36	0.29–6.51	0.699
American Indian/Alaska Native	1.32	0.17–9.96	0.790	–	–	–
Grade						
Well differentiated; Grade I	1.00			–	–	–
Moderately differentiated; Grade II	1.87	0.44–7.73	0.389	–	–	–
Poorly differentiated; Grade III	1.30	0.37–4.61	0.681	–	–	–
Undifferentiated; anaplastic; Grade IV	2.12	0.60–7.45	0.242	–	–	–
SEER summary stage						
Localized	1.00			1.00		
Regional	1.23	0.76–2.01	0.401	0.68	0.18–2.57	0.683
Distant	1.92	1.19–3.10	0.008	1.06	0.29–3.90	0.934
Surgery						
Surgery not performed	1.00			1.00		
Surgery performed	0.50	0.26–0.96	0.036	0.43	0.14–1.32	0.138
Radiation						
Radiation not received	1.00			1.00		
Radiation received	0.394	0.16–1.00	0.049	1.37	0.38–4.90	0.629
Chemotherapy						
Chemotherapy not received	1.00			1.00		
Chemotherapy received	0.33	0.21–0.51	<0.001	1.79	0.68–4.71	0.238

Abbreviations: HA, hemangiosarcoma; HEHE, hepatic epithelioid hemangioendothelioma.

## 4 DISCUSSION

This study represents the most detailed analysis and largest cohort of patients diagnosed with HA and HEHE to date. We found that HA are more prevalent in White males in their sixth decade of life. Moreover, most of these tumors are histologically undifferentiated and present with distant metastasis at the time of diagnosis. Overall, HA represents an aggressive MVT in comparison to HEHE.

A study by Yasir et al. studying 80 cases of angiosarcomas reported significant inter‐tumor heterogeneity and morphological patterns within angiosarcomas. These varied patterns may contribute to their distinct biological behavior and makes diagnosis challenging. About 14% of patients with HA had surgery, 31% received chemotherapy, and only 5% had radiation therapy. Moreover, from those few cases where radiation therapy was employed, HA proved to be a radiation‐resistant tumor.^26^

For HA, surgery provided 8 months median overall survival compared to 2 months overall survival when patients did not receive surgical intervention. These are partly explained due to the incidence of surgical complications such as intraperitoneal hemorrhage in these highly vascularized tumors. These observations are concordant with previous studies on MVL.^2^, ^5^, ^27^, ^28^ Moreover, Li et al. performed a systemic literature review involving 186 patients with liver sarcomas and concluded that partial hepatectomy as a surgical procedure could give a survival advantage in tumors smaller than 10 cm.^11^, ^12^, ^29^ Altogether, HA remains an aggressive tumor despite intervention. Tumor markers and genome‐wide studies should be explored to have better patient stratification and understanding of treatment response.^9^ Additionally, standardized treatment guidelines are in need. The aggressiveness and poor outcome of this tumor, demands for multicentric efforts to identify better clinical management and effective therapeutic strategies.

Unlike HA, HEHE has a predilection for females. Most patients are White and are diagnosed in their 50s. Our findings are concordant with previous literature which reported slight predominance in females and middle‐aged adults.^30^ Most HEHE tumors are presented with distant metastases. Unlike HA, most patients with HEHE tumors have a poor survival advantage from surgery. On a study performed on HEHE by Noh O, et al. treatment modalities used throughout the past 30 years were analyzed. They divided their results in decades, concluding that despite of the increase in surgical interventions, HEHE outcomes have remained the same.^23^

Survival of HEHE is higher compared to HA with a median survival of 182 and 2 months, respectively. Pregliasco et al. reported 1‐ and 5‐year overall survival of 80% and 64% for HEHE, a slightly higher survival in these patients compared to the current study (72% and 59%).^13^ Differences reported in overall survival in cases of HEHE may be accounting for distinct subsets of tumors and maybe identifying patients treated with different modalities. Our research further supports that HEHE has a slow indolent progression commonly associated with a better overall prognosis when compared to HA.

This is a retrospective study with limitations of such an analysis. The SEER database is a vast composite of cancers across the United States; however, SEER lacks central review by an expert pathologist. Within the studied tumors, unknown tumor grade accounted for 74.3% of HA cases and 82.5% of HEHE cases. The histopathological differences among these tumors could be an important variable for better patient stratification and treatment selection. For instance, other MVTs, such as hemangiopericytomas and angiomyolipomas of the liver, have few or no available data to warrant analysis.

Additionally, SEER lacks detailed information about chemotherapy regimens, transcatheter arterial chemoembolization (TACE), liver transplantation, and medical co‐morbid conditions which may have a bearing on clinical outcomes and therapeutic interventions. This is in line with the lack of studies discussing different treatment modalities. One of the largest studies, was performed by Cardinal et al. on a 25‐patient single institution study. The group characterized the treatments utilized for EHE. Liver transplantation was the preferred modality (68%) for locally advanced EHE, whereas TACE (16%) was reserved for patients with disseminated disease or comorbidities that were not suitable to undergo LT. Resection and the combination of TACE followed by LT, were both only utilized in 8% of cases.^31^

In conclusion, MVTs are a rare subset of hepatic tumors with wide clinical presentation and aggressiveness. Aside from imaging features, we observed that patients’ demographics such as race, age, and gender can help to elucidate distinct subtypes of MVT. Within MVTs, standardized treatment guidelines are in need. The documentations of tumor grade and treatment modalities are absent variables in most HA and HEHE cases. Further studies are needed to deepen our knowledge about the histopathology of these tumors, the outcomes of liver transplantation as a therapeutic alternative, and available molecular targets for MVTs.

## ETHICS APPROVAL AND CONSENT TO PARTICIPATE

Not indicated as this article only used histopathological images that are completely unidentifiable and there is no information on the individual whose case is presented.

## CONFLICT OF INTEREST

The authors declare no conflict of interest.

## Data Availability

The data generated and analyzed during the current study are available from Surveillance, Epidemiology, and End Results (SEER) Program. [https://seer.cancer.gov.]
